# Identification of a Novel Oxidative Stress-Based Molecular Classification and Treatment Vulnerabilities in WHO Grade 2/3 Meningiomas

**DOI:** 10.32604/or.2025.066308

**Published:** 2025-09-26

**Authors:** Xiao-Xiao Luo, Bi Peng, Jian-Hua Wang, Guang-Yuan Hu, Xiang-Lin Yuan, Guo-Xian Long

**Affiliations:** Department of Oncology, Tongji Hospital, Tongji Medical College, Huazhong University of Science and Technology, Wuhan, 30030, China

**Keywords:** Meningiomas, oxidative stress, classification, Forkhead Box M1 (FOXM1), Prion Protein (PRNP)

## Abstract

**Objective:**

The World Health Organization (WHO) grading based on histopathology cannot always accurately predict tumor behavior of meningiomas. To overcome the limitations of the WHO grading, the study aims to propose a novel oxidative stress-based molecular classification for WHO grade 2/3 meningiomas.

**Methods:**

Differentially expressed oxidative stress-related genes were analyzed between 86 WHO grade 1 (low grade) meningiomas and 99 grade 2/3 (high grade) meningiomas. An oxidative stress-based molecular classification was developed in high-grade meningiomas through consensus clustering analysis. Immune microenvironment features, responses to immunotherapy and chemotherapy, and targeted drugs were evaluated. Three machine learning models: logistic regression, support vector machine, and random forest, were built for differentiating the classification. Key oxidative stress-related genes were verified in human meningeal cells (HMC) and two meningioma cells (CH-157MN and IOMM-Lee) via reverse transcription quantitative polymerase chain reaction (RT-qPCR) and western blot. After knockdown of Forkhead Box M1 (FOXM1) or Prion Protein (PRNP), cell growth, migration, and reactive oxygen species (ROS) levels were measured through cell counting kit-8 (CCK-8), transwell, and immunofluorescence, respectively.

**Results:**

We classified high-grade meningiomas into two oxidative stress-based clusters, termed cluster 1 and cluster 2. Cluster 1 exhibited higher infiltrations of immune and stromal cells and higher expression of classic immune checkpoints: Cluster of Differentiation 86 (CD86), Programmed Cell Death 1 (PDCD1), and Leukocyte-Associated Immunoglobulin-Like Receptor 1 (LAIR1), indicating that cluster 1 meningiomas might respond to immunotherapy. Drug sensitivity was heterogeneous between the two clusters. Three classifiers were established, which could accurately differentiate this molecular classification. FOXM1 and PRNP were experimentally evidenced to be highly expressed in meningioma cells, and their knockdown hindered cell growth and migration and triggered ROS accumulation.

**Conclusion:**

In summary, our findings established a novel oxidative stress-based molecular classification and identified potential treatment vulnerabilities in high-grade meningiomas, which might assist personalized clinical management.

## 1 Introduction

Meningiomas are the most prevalent primary intracranial tumors, which account for approximately 50% of all nonmalignant tumors in adults [1]. Currently, clinical management of meningiomas is guided by the World Health Organization (WHO) grading, a three-tiered grading system based on histopathological characteristics. Grade 2 and 3 (high-grade) meningiomas exhibit stronger aggressiveness, and patient prognosis after surgery alone is still unsatisfactory [2]. The WHO grading remains the main measure for predicting prognostic outcomes and guiding postoperative treatment decisions [3]. Nevertheless, clinical manifestations may sometimes not fully conform to the WHO grading [4–6]. For instance, despite aggressive therapy, a few patients with histologically benign meningiomas develop repeated relapses, and those with higher grade remain stable after surgical resection. Incomplete tumor sampling, inter-observer variability in histological evaluation, and ambiguous histological characteristics all pose challenges and result in uncertainty in disease management [4–6]. Moreover, the possibility that malignant potential may not be uniformly reflected in assessing histological characteristics [7]. More prognostic information is thus required for optimizing patient management [8]. Reliable and accessible predictors of prognostic outcomes after diagnosis are urgently needed.

The brain accounts for ~20% of the body’s total metabolic activity and consumes more oxygen than other tissues, suggesting the possibility of more free radical production than other organs [9,10]. Hence, oxidative stress is an important disruptor of normal brain homeostasis and participates in brain tumorigenesis [11]. Due to the brain’s high metabolic activity and susceptibility to the damage of reactive oxygen species (ROS), brain tumor patients have been found to display increased oxidative damage [12,13]. Oxidative stress event is implicated in the malignant behaviors of brain tumor cells and drug resistance [14–17]. Based on the importance of oxidative stress in brain tumors, we reported an oxidative stress-based molecular classification in high-grade meningiomas for assisting clinical decision making and identified the two key oxidative stress-related genes FOXM1 and PRNP as potential treatment vulnerabilities.

## 2 Materials and Methods

### 2.1 Meningioma Transcriptome Data

From the GSE183653 dataset in the Gene Expression Omnibus (https://www.ncbi.nlm.nih.gov/geo/query/acc.cgi?acc=GSE183653, accessed on 17 August 2025), RNA sequencing (RNA-seq) profiles of 185 meningioma patients were downloaded [1,18,19]. The dataset contained 86 WHO grade 1 (low grade) and 99 grade 2/3 (high grade) meningiomas (Supplementary Table S1) based on the GPL20301 Illumina HiSeq 4000 platform. Furthermore, we gathered the transcriptome data of 42 high-grade meningioma patients from the GSE74385 dataset (https://www.ncbi.nlm.nih.gov/geo/query/acc.cgi?acc=GSE74385, accessed on 17 August 2025) as an external validation cohort [20].

### 2.2 Differential Expression Analysis

Differentially expressed genes between grade 1 and grade 2/3 meningiomas were selected by the ‘limma’ package (version 3.50.0) [21]. The threshold was set as |log_2_fold change| > 0.585 and *p* < 0.05. The oxidative stress-related gene set (Supplementary Table S2) was gathered from prior literature [22,23]. Differentially expressed oxidative stress-related genes were subsequently acquired, which were then visualized into volcano diagram and heatmap.

### 2.3 Functional Enrichment Analysis

Gene Ontology (GO) biological processes and Kyoto Encyclopedia of Genes and Genomes (KEGG) pathways were analyzed by the ‘clusterProfiler’ package (version 4.4.0) [24]. The hallmark gene set of “h.all.v7.4.entrez.gmt” was acquired from the Molecular Signatures Database (https://www.gsea-msigdb.org/gsea/msigdb) (accessed on 17 August 2025) [25], which was analyzed by the ‘GSVA’ package (version 1.34.0) [26]. Upset plots and heatmap were established to visualize the functional enrichment results.

### 2.4 Consensus Clustering Analysis

Based on the oxidative stress-related genes with differential expression, consensus clustering analysis was carried out by the ‘ConsensusClusterPlus’ package (version 1.73.0) [27]. The optimal value was determined based on the expression profiles of oxidative stress-related gene, and consensus matrix of meningioma samples at the optimal value was shown in heatmap. This classification was confirmed through principal component analysis (PCA).

### 2.5 Immune Microenvironment Estimation

Infiltration levels of 22 immune cell compositions were quantified based on transcriptome profiling by the CIBERSORT package (version 0.1.0) [28], which were visualized into stacked bar chart. In addition, tumor purity and stromal and immune cell admixture were inferred by the ESTIMATE package (version 1.0.13) [29]. The infiltration levels of immune cell types between the two clusters were compared and shown in box plots. The relationships between feature genes and immune cell infiltrations were analyzed and visualized using lollipop chart.

### 2.6 Drug Sensitivity Evaluation

Based on the Genomics of Drug Sensitivity in Cancer 2.0 database (https://www.cancerrxgene.org/) (accessed on 17 August 2025) [30], IC_50_ values of drugs were estimated by the ‘oncoPredict’ package (version 1.2) [31].

### 2.7 Development of Classifiers and Selection of Feature Genes

The FindCorrelation function in the ‘caret’ package (version 7.0-1) was adopted to identify and eliminate genes with high expression correlation, and the ‘rfe’ package (version 7.0-1) was utilized to further identify genes with greater contribution to meningioma clusters. Then, the createDataPartition function was used to split meningiomas into training and test sets. In the training set, using the ‘caret’ package (version 7.0-1), three classification algorithms: logistic regression (LogitBoost), linear kernel support vector machine (SVM), and random forest (randomforest) were conducted for model training. Finally, the classification results were predicted with the predict function. Receiver operator characteristic curve (ROC) analysis was implemented by the ‘pROC’ package (version 1.17.0.1), and the area under the curve (AUC) was calculated. Feature genes were selected through any two algorithms (LogitBoost, random forest, and SVM), and the results were visualized using Venn diagram.

### 2.8 Cell Culture

A human meningeal cell line (HMC) and two WHO grade 2/3 meningioma cell lines (CH-157MN and IOMM-Lee) were acquired from the Shanghai Institute of Cell Research (Shanghai, China). All the cells were cultivated in Dulbecco’s Modified Eagle’s Medium (DMEM; HyClone, Logan, UT, USA) containing 10% fetal bovine serum (FBS; HyClone) and 1% penicillin/streptomycin (HyClone) in a humidified environment with 5% CO_2_ at 37°C. All cell lines were subjected to short tandem repeat (STR) profiling and mycoplasma infection testing to authenticate their real identities without contamination.

### 2.9 Reverse Transcription Quantitative Polymerase Chain Reaction (RT-qPCR)

Total RNA was isolated from HMC, CH-157MN, and IOMM-Lee cells by High Pure RNA Isolation Kit (11828665001, Roche, Basel, Switzerland), with subsequent cDNA synthesis. The cDNA products were then assessed by SsoFast EvaGreen Supermix (1725205, Bio-Rad, Hercules, CA, USA) and CFX96 Touch Real-Time PCR Detection System (Bio-Rad). Supplementary Table S3 summarizes the primer sequences of human *AOX1, FOXM1, GPX3, PRNP, SEPP1*, and *GAPDH*. The relative mRNA expression was analyzed through the 2^−ΔΔCt^ method.

### 2.10 Western Blot and Antibodies

HMC, CH-157MN, and IOMM-Lee cells were lysed by RIPA lysis (R0010, Solarbio, Beijing, China). Total proteins were quantified by BCA Protein Assay Kit (PC0020, Solarbio), as previously described [32]. The 20~40 μg proteins were subjected to 10%~12% SDS-PAGE gels and electroblotted to PVDF membranes (Bio-Rad). Subsequently, the membranes were probed with primary antibody against FOXM1 (1/1000; ab207298), PRNP (1/5000; ab52604), or SEPP1 (1/1000; ab277526), followed by incubation with goat anti-rabbit IgG H&L (HRP) preadsorbed secondary antibody (1/10,000; ab7090). All the antibodies were from Abcam (Boston, MA, USA). The membranes were re-probed with GAPDH (1/10,000; ab181602) to ensure an equal amount of protein load. Protein bands were developed using ECL kit (PE0010, Solarbio) following the manufacturer’s instructions.

### 2.11 Transient Transfection

CH-157MN and IOMM-Lee cells were cultured in 6-well plates until they reached 70%–80% confluence. Specific small interfering RNAs (siRNAs) of *FOXM1* or *PRNP* were transiently transfected into CH-157MN and IOMM-Lee cells by Lipofectamine 3000 (Invitrogen, Carlsbad, CA, USA) in accordance with the manufacturer’s instructions. The siRNA sequences were as follows: *FOXM1* siRNA, 5^′^-UCAACAGCACUGAGAGGAA-3^′^ (sense), 5^′^-UUCCUCUCAGUGCUGUUGA-3^′^ (antisense); *PRNP* siRNA, 5^′^-AGUGGAACAAGCCGAGUAA-3^′^ (sense), 5^′^-UUACUCGGCUUGUUCCACU-3^′^ (anti- sense); negative control (NC) siRNA, 5^′^-UUCUCCGAACGUGUCACGUTT-3^′^ (sense), 5^′^-ACGUGACA- CGUUCGGAGAATT-3^′^ (antisense). After transfection for 48 h, the cells were used for subsequent experiments.

### 2.12 Cell Viability Assay

Cell viability of CH-157MN and IOMM-Lee cells was assessed by Cell Counting Kit-8 (CCK-8) kit (CK04, DOJINDO, Kumamoto, Japan) following the manufacturer’s instructions. CH-157MN and IOMM-Lee cells were seeded into a 96-well plate (2 × 10^3^ cells/well). After 48 h, the cells were treated with CCK-8 reagent (10 μL/well) for 2 h. The absorbance at 450 nm was measured by a microplate reader (SpectraMAX M2, Molecular Devices, San Jose, CA, USA).

### 2.13 Transwell Assay

Cell migration of CH-157MN and IOMM-Lee cells was evaluated by transwell chamber (Corning, NY, USA). As previously described [33], cells were inoculated in a serum-free medium in the upper insert of the Transwell system. The lower insert was filled with culture medium. Following 24-h incubation, the cells were fixed with 100% methanol and stained with 0.5% crystal violet. Finally, migrated cells were counted under a light microscope (Axiovert 200, Zeiss, Jena, Germany).

### 2.14 Immunofluorescence

CH-157MN, and IOMM-Lee cells were inoculated in a 6-well plate (2 × 10^5^ cells/well). Intracellular ROS assay was implemented with fluorescent probe 2,7-dichlorofluorescein diacetate (DCFH-DA) (Sigma-Aldrich, St. Louis, MO, USA). Briefly, the cells were probed with DCFH-DA for 30 min. The ROS fluorescence was assessed under a fluorescence microscope (Zeiss, Jena, Germany).

### 2.15 Statistical Analysis

All the analyses were achieved by R v4.2.1 and GraphPad Prism v9.0.1 (GraphPad Software, San Diego, CA, USA). Comparison between groups was analyzed through Student’s *t*-test or one-way ANOVA. Correlation analysis was conducted through the Pearson or Spearman test. Statistical significance was established at *p* < 0.05.

## 3 Results

### 3.1 Development of an Oxidative Stress-Based Molecular Classification for High-Grade Meningiomas

**Figure 1:**
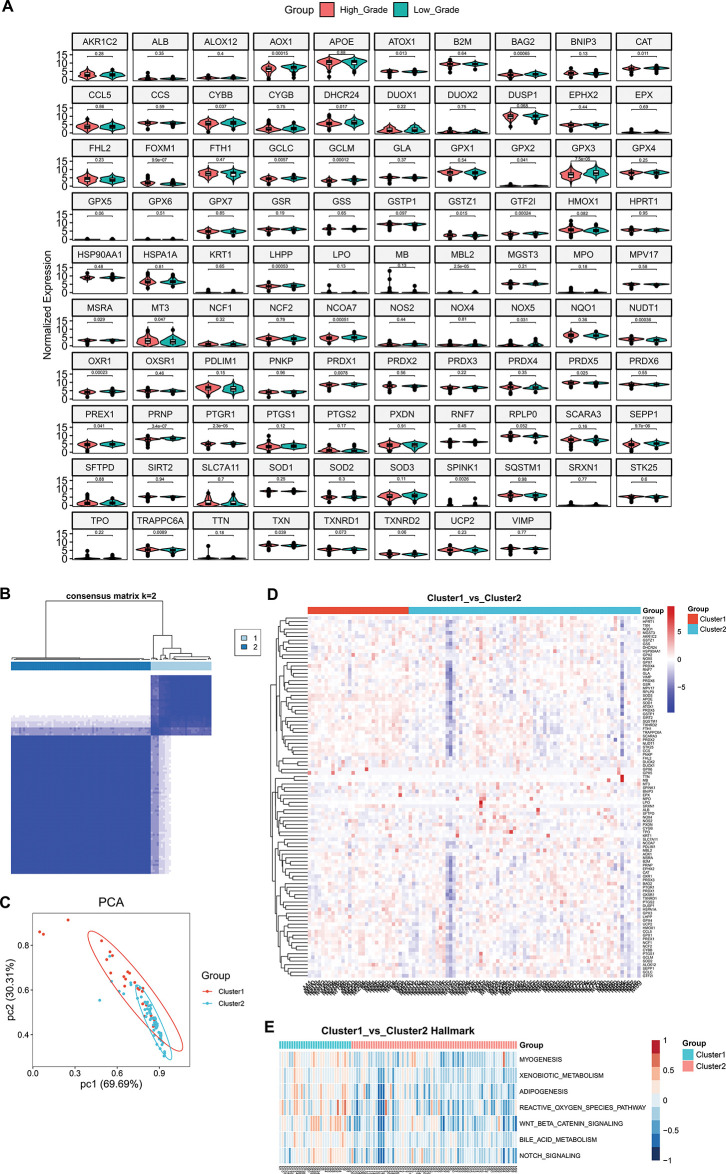
Development of an oxidative stress-based molecular classification for meningiomas. (**A**) Violin diagrams show differential expression of oxidative stress-related genes in high-grade and low-grade meningiomas. (**B**) Heatmap shows consensus matrix of meningioma samples at the optimal value (*k* = 2) based on the expression profiles of oxidative stress-related genes. The rows and columns of the consensus matrix represent meningioma samples. The values of the consensus matrix range from 0 (impossible to cluster together) to 1 (always clustered together) in white to dark blue. (**C**) PCA plots illustrate the different expression profiles of oxidative stress-related genes between the two clusters. Each dot represents one sample. (**D**) Heatmap shows the expression of oxidative stress-related genes in two clusters. Red, up-regulated genes; blue, down-regulated genes. (**E**) Heatmap shows the differential hallmark pathways with *p*-value ≤ 0.05 and |*t*-value| ≥ 4 between two clusters. Low to high GSVA scores are colored from blue to red

In the present study, 189 genes displayed up-regulation in high-grade meningiomas vs. low-grade meningiomas, with 737 displaying down-regulation (Supplementary Fig. S1A–C). The up-regulated genes were primarily linked with biosynthetic and metabolic processes (Supplementary Fig. S1D), while the down-regulated genes were mainly connected to nervous system development (Supplementary Fig. S1E). In addition, it was found that the differentially expressed genes presented the associations with oncogenic pathways (e.g., cell cycle, cellular senescence, p53, mitogen-activated protein kinases (MAPK), phosphoinositol-3 kinase (PI3K)-Akt, Ras, Ras-associated protein1 (Rap1), cyclic adenosine monophosphate (cAMP), and Forkhead box O (FoxO) signaling pathways, and pathways in cancers) (Supplementary Fig. S1F,G). These findings uncovered the biological significance of the differentially expressed genes in high-grade meningiomas. Especially, we gathered 99 oxidative stress-related genes from a previously published article [34]. Among them, 98 genes were detected in the meningioma samples. It was noted that most of the oxidative stress-related genes presented differential expression between high-grade and low-grade meningiomas (Fig. 1A), especially AOX1, FOXM1, GPX3, PRNP, and SEPP1. Based on the oxidative stress-related genes with differential expression, two oxidative stress-based molecular clusters were identified for high-grade meningiomas (Fig. 1B; Supplementary Table S4), namely cluster 1 (*n* = 30) and cluster 2 (*n* = 69). The accuracy in the molecular classification was proven by PCA (Fig. 1C). Furthermore, we adopted the GSE74385 dataset (including 42 high-grade meningioma samples) for external validation. Our results confirmed that the oxidative stress-based molecular classification was robust (Supplementary Fig. S2). As illustrated in Fig. 1D, the oxidative stress-related genes exhibited higher expression in cluster 1 versus cluster 2, reflecting the higher activity of oxidative stress in cluster 1. Among classic hallmarks, myogenesis, adipogenesis, xenobiotic metabolism, bile acid metabolism, ROS pathway, Wnt/beta-catenin signaling, and Notch signaling were notably activated in cluster 1 (Fig. 1E).

### 3.2 Heterogeneous Immune Microenvironment and Immunotherapy Responses of Two Oxidative Stress-Based Clusters

**Figure 2:**
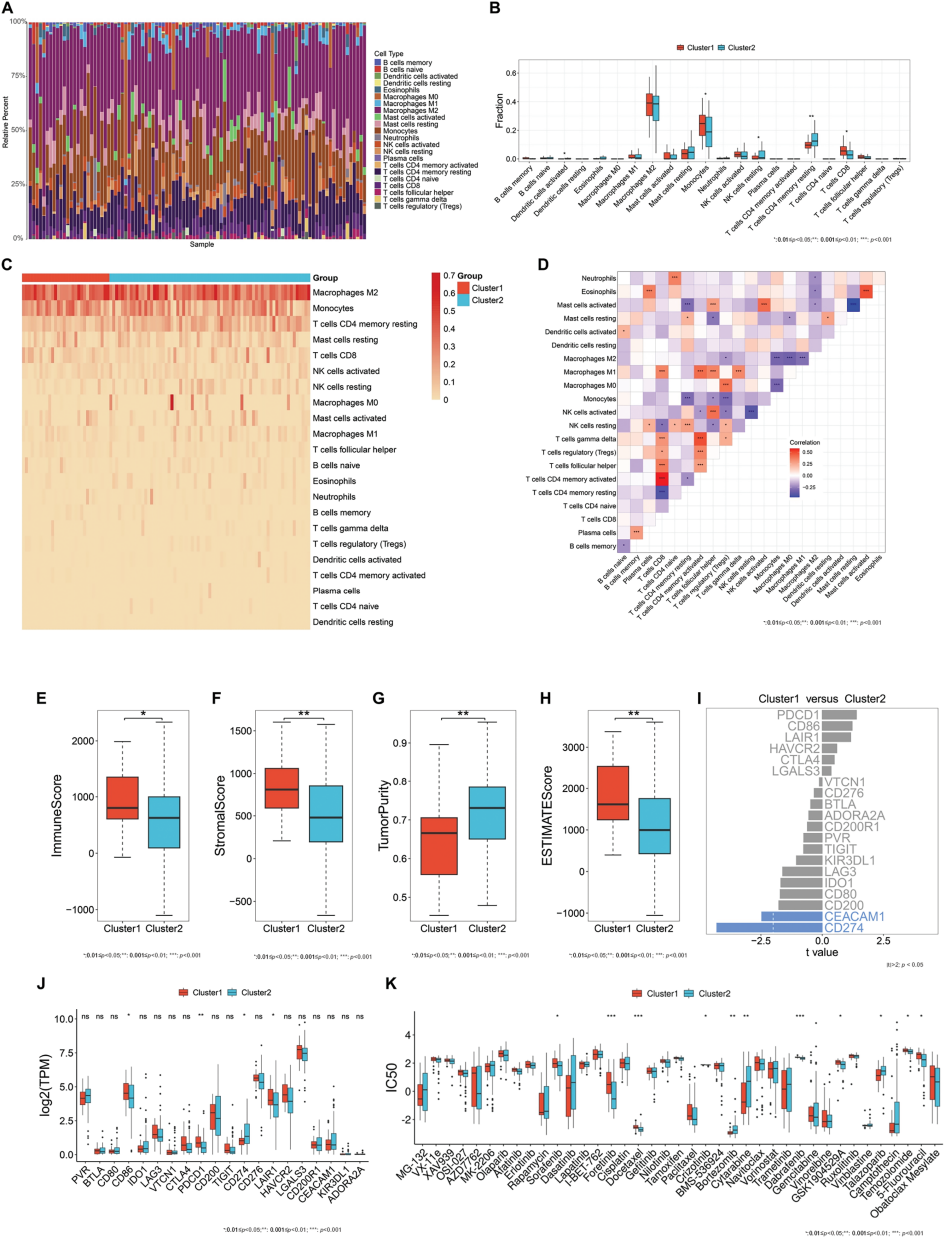
Heterogeneous immune microenvironment, immunotherapy responses, and drug sensitivity in two oxidative stress-based clusters. (**A**) A stacked bar chart displays the relative infiltration levels of 22 immune cell types across meningioma tissues. Each immune cell type is labeled with a unique color. The sum of the relative infiltration levels of the immune cell types in each sample is 100%. (**B**) Box plots show the differential infiltration levels of immune cell types between the two clusters. (**C**) The heatmap illustrates the relative infiltration levels of immune cell types across two clusters. The darker the color, the higher the infiltration levels. (**D**) Heatmap visualizes the relationships between different immune cell types across meningiomas. Red, positive correlation; blue, negative correlation. (**E**–**H**) Box plots display the differential immune and stromal scores, tumor purity, and ESTIMATE score in two clusters. (**I**) Comparison of the expression of immune checkpoints between the two clusters. The differentially expressed immune checkpoints with *p*-value < 0.05 and |*t*-value| > 2 are colored blue. (**J**) Box plots exhibit the differential expression of immune checkpoints between clusters. (**K**) Box plots exhibit the differential IC_50_ values of bortezomib, crizotinib, cytarabine, dabrafenib, docetaxel, foretinib, GSK1904529A, sorafenib, talazoparib, temozolomide, and 5-fluorouracil between two clusters. *p-value < 0.05; ^**^p-value < 0.01; ***p-value < 0.001; ns: *p*-value > 0.05

Immune cell compositions in the immune microenvironment were then estimated in high-grade meningiomas. As depicted in Fig. 2A, the infiltration levels of the immune cell compositions were remarkably heterogeneous among patients with high-grade meningiomas. It was also found that there were higher infiltrations of activated dendritic cells, resting natural killer (NK) cells, and memory resting CD4^+^ T cells as well as lower infiltrations of monocytes, and CD8^+^ T cells in cluster 2 in comparison to cluster 1 meningiomas (Fig. 2B,C), revealing the heterogeneity in the immune microenvironment between two oxidative stress-based clusters. Additionally, there were complex interactions between immune cell compositions in high-grade meningiomas (Fig. 2D).

Infiltrating immune and stromal cells and tumor purity were inferred in high-grade meningiomas. Cluster 1 meningiomas exhibited higher immune, stromal, and ESTIMATE scores and lower tumor purity in comparison to cluster 2 meningiomas (Fig. 2E–H). Additionally, classic immune checkpoints: Cluster of Differentiation 86 (CD86), Programmed Cell Death 1 (PDCD1), and Leukocyte-Associated Immunoglobulin-Like Receptor 1 (LAIR1) had higher expression in cluster 1 (Fig. 2I,J).

### 3.3 Heterogeneity in Drug Sensitivity between Two Oxidative Stress-Based Clusters

Bortezomib, cytarabine, and talazoparib showed the lower IC50 values in cluster 1 meningiomas, with the lower IC_50_ values of crizotinib, dabrafenib, docetaxel, foretinib, GSK1904529A, sorafenib, temozolomide, and 5-fluorouracil in cluster 2 meningiomas (Fig. 2K). It was thus inferred that cluster 1 meningiomas were more sensitive to bortezomib, cytarabine, and talazoparib, while cluster 2 meningiomas displayed higher sensitivity to crizotinib, dabrafenib, docetaxel, foretinib, GSK1904529A, sorafenib, temozolomide, and 5-fluorouracil.

### 3.4 Construction of Three Machine Learning Models for Differentiating the Oxidative Stress-Based Molecular Classification

**Figure 3:**
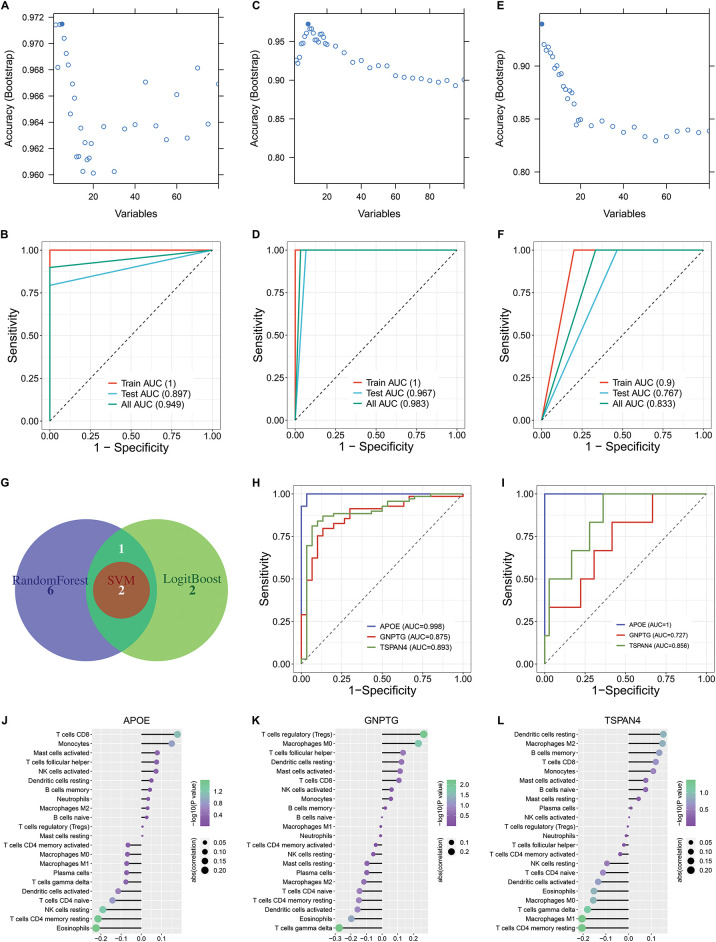
Construction of three machine learning models for differentiating the oxidative stress-based molecular classification and selection of feature genes through integrative machine learning algorithms. (**A**) Selection of genes contributing more to the oxidative stress-based molecular classification by LogitBoost algorithm: APOE, TSPAN4, TMEM223, DHRS4, and GNPTG. (**B**) ROC curves of the LogitBoost model in differentiating between two clusters in the training set, test set, and total set. (**C**) Selection of genes contributing more to the classification by the random forest algorithm: APOE, TSPAN4, GNPTG, CYBA, VMO1, SELM, C1orf56, CMTM2, and LINC00116. (**D**) ROC curves of the random forest model in the training set, test set, and total set. (**E**) Selection of genes contributing more to the classification by the support vector machine (SVM) algorithm: APOE and TSPAN4. (**F**) ROC curves of the SVM model in the training set, test set, and total set. (**G**) A Venn diagram shows the common feature genes by any two algorithms (LogitBoost, random forest, and SVM): APOE, GNPTG, and TSPAN4. (**H**) ROC curves of APOE, GNPTG, and TSPAN4 in differentiating two oxidative stress-based clusters. (**I**) External validation of the performance of the above feature genes in differentiating the two oxidative stress-based clusters in the GSE74385 dataset. (**J**–**L**) Lollipop charts illustrate the relationships between (**J**) APOE, (**K**) GNPTG, and (**L**) TSPAN4 and immune cell infiltrations across meningiomas

To facilitate the clinical application of the oxidative stress-based molecular classification, three machine learning computational approaches were adopted for differentiating two clusters. Genes contributing more to the classification were selected via LogitBoost (APOE, TSPAN4, TMEM223, DHRS4, and GNPTG), randomforest (APOE, TSPAN4, GNPTG, CYBA, VMO1, SELM, C1orf56, CMTM2, and LINC00116), and SVM (APOE and TSPAN4), respectively (Fig. 3A–F). In addition, ROC analysis proved the accuracy of the LogitBoost, randomforest, and SVM models in differentiating the oxidative stress-based molecular classification in the training, test, and total sets (Fig. 3A–F).

### 3.5 Selection of Feature Genes through Integrative Machine Learning Algorithms

The feature genes: APOE, GNPTG, and TSPAN4 were then selected in accordance with the results from any two algorithms (LogitBoost, random forest, and SVM) (Fig. 3G). ROC analysis proved that the feature genes accurately differentiated the two oxidative stress-based clusters (Fig. 3H). The excellent performance of the feature genes in differentiating the two oxidative stress-based clusters was externally validated in the independent cohort (GSE74385) (Fig. 3I). All the feature genes exhibited remarkable interactions with infiltrations of immune cell compositions across high-grade meningiomas (Fig. 3J–L).

### 3.6 Experimental Verification of the Key Oxidative Stress-Related Genes in Meningioma Cells

**Figure 4:**
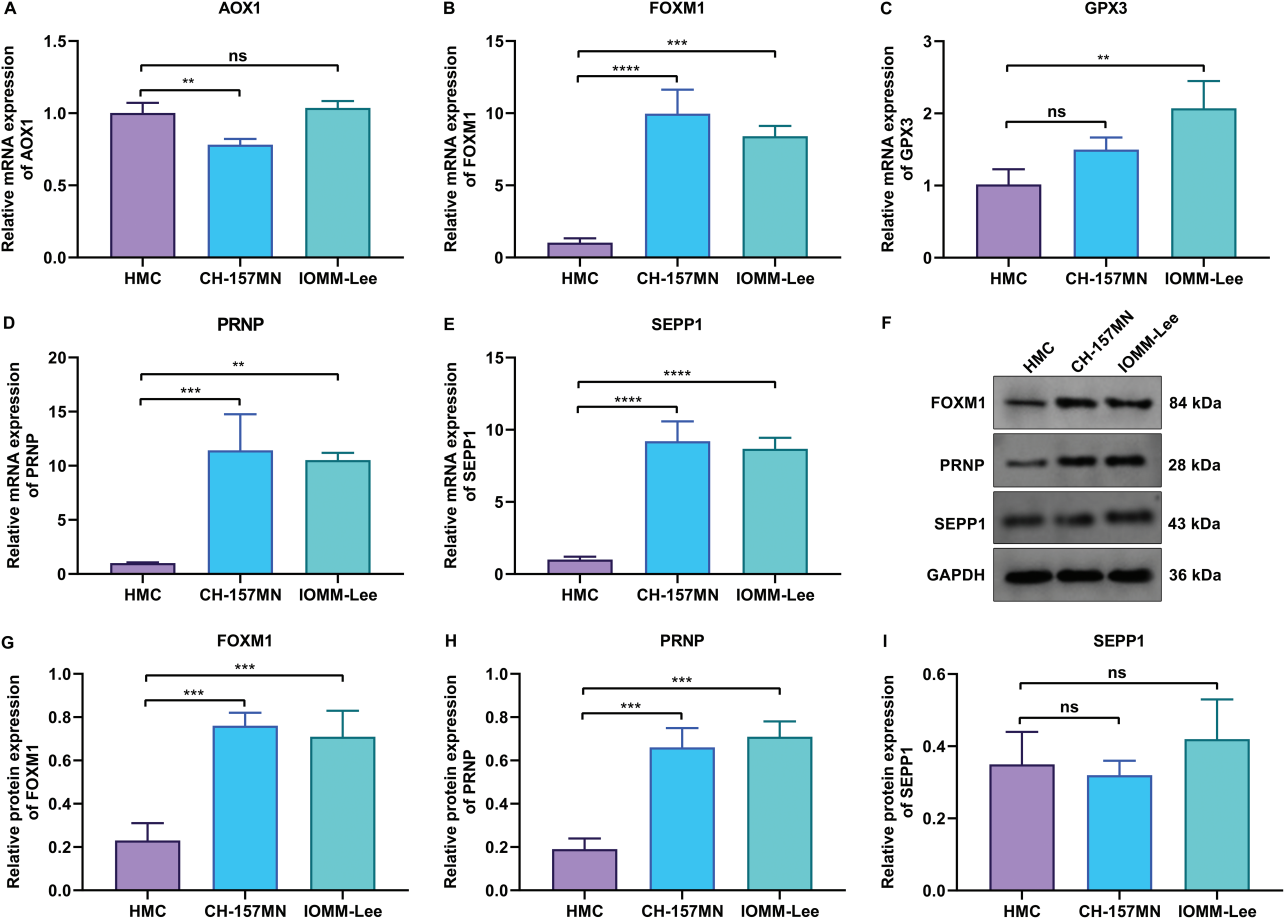
Experimental verification of the abnormal expression of key oxidative stress-related genes in meningioma cells. (**A**–**E**) RT-qPCR for detecting the mRNA expression levels of (**A**) AOX1, (**B**) FOXM1, (**C**) GPX3, (**D**) PRNP, and (**E**) SEPP1 in human meningeal cells (HMC), and human meningioma cells (CH-157MN, and IOMM-Lee). (**F**) Western blot results of FOXM1, PRNP, and SEPP1 in HMC, CH-157MN, and IOMM-Lee cells. (**G**–**I**) Quantification of the protein expression levels of (**G**) FOXM1, (**H**) PRNP, and (**I**) SEPP1 in HMC, CH-157MN, and IOMM-Lee cells. **p-value < 0.01; ***p-value < 0.001; ****p-value < 0.0001; ns: *p*-value > 0.05

This study further experimentally validated the key oxidative stress-related genes: AOX1, FOXM1, GPX3, PRNP, and SEPP1 in human meningeal cells (HMC) and two meningioma cells (CH-157MN and IOMM-Lee). RT-qPCR analysis showed that AOX1 expression was significantly lower in CH-157MN cells not IOMM-Lee cells vs. HMC cells (Fig. 4A). FOXM1, PRNP, and SEPP1 exhibited higher expression in CH-157MN and IOMM-Lee cells vs. HMC cells, while GPX3 displayed higher expression in IOMM-Lee cells not CH-157MN cells in comparison to HMC cells (Fig. 4B–E). Further western blot proved the higher expression of FOXM1 and PRNP in two meningioma cells vs. HMC cells (Fig. 4F–H and Supplementary Fig. S3), without significant difference in SEPP1 expression (Fig. 4I). Altogether, among the key oxidative stress-related genes, FOXM1 and PRNP were experimentally evidenced to be up-regulated in meningiomas.

### 3.7 Knockdown of FOXM1 and PRNP Inhibits the Proliferation and Migration of Meningioma Cells

**Figure 5:**
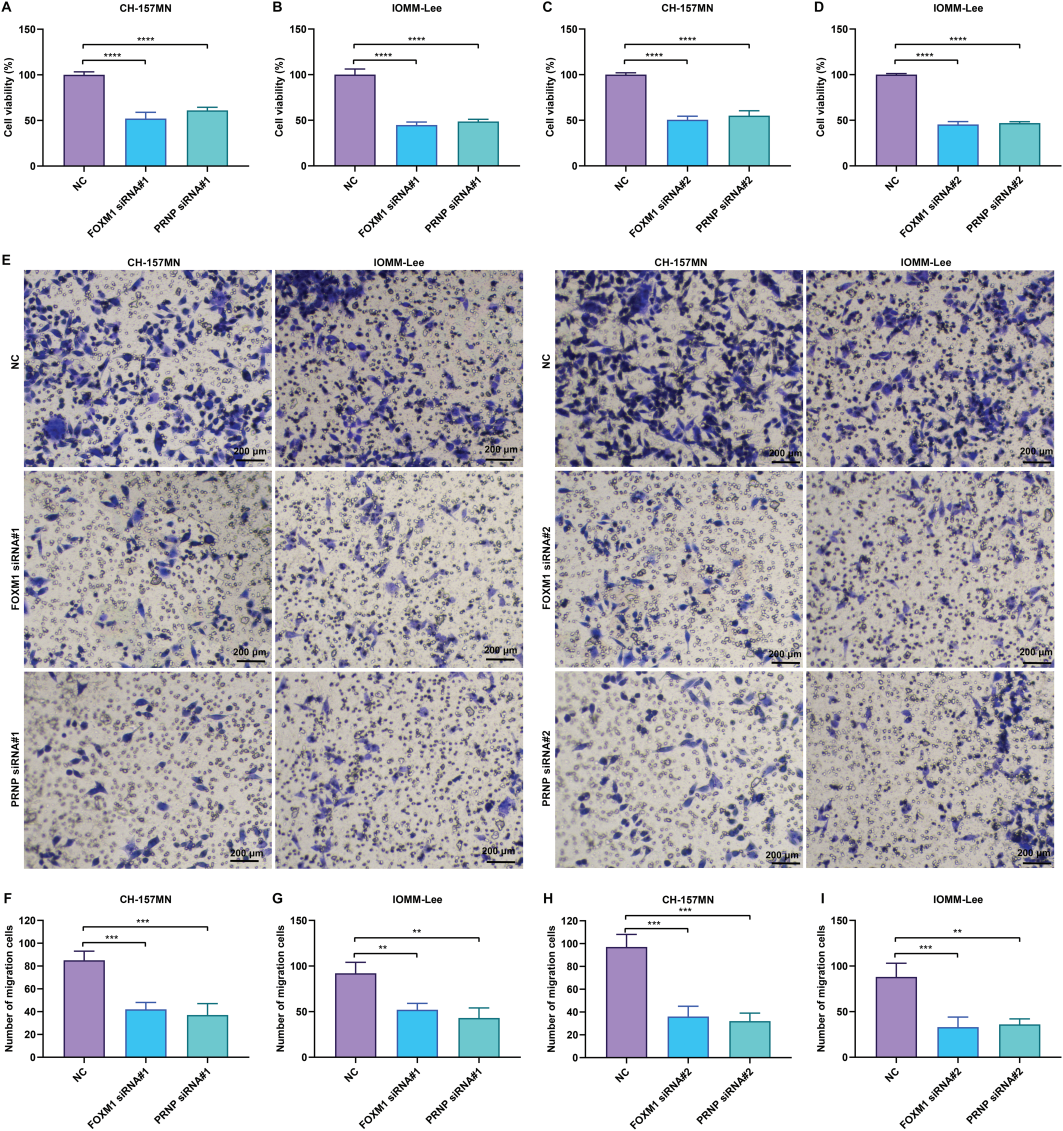
Knockdown of FOXM1 or PRNP inhibits the viability and migration of meningioma cells. **(A**–**D**) CCK-8 assay for evaluating the viability of CH-157MN and IOMM-Lee cells transfected with FOXM1 siRNA#1/#2 or PRNP siRNA#1/#2. (**E**–**I**) Transwell assay for assessing the migration of CH-157MN and IOMM-Lee cells in the context of FOXM1 siRNA#1/#2 or PRNP siRNA#1/#2 transfection. Scale bar, 200 μm. **p-value < 0.01; ***p-value < 0.001; ****p-value < 0.0001

To verify the biological roles of FOXM1 and PRNP, two specific siRNAs were transiently transfected into CH-157MN and IOMM-Lee meningioma cells to silence their expression, respectively. CCK-8 analysis showed that the viability of FOXM1- or PRNP-silenced CH-157MN and IOMM-Lee cells was prominently impaired (Fig. 5A–D). From the results of the transwell assay, migration of CH-157MN and IOMM-Lee cells was notably attenuated in the context of suppression of FOXM1 and PRNP (Fig. 5E–I). Hence, the silence of FOXM1 and PRNP lowered the proliferation and migration of meningioma cells.

### 3.8 Knockdown of FOXM1 and PRNP Induces the Excessive Accumulation of ROS in Meningioma Cells

**Figure 6:**
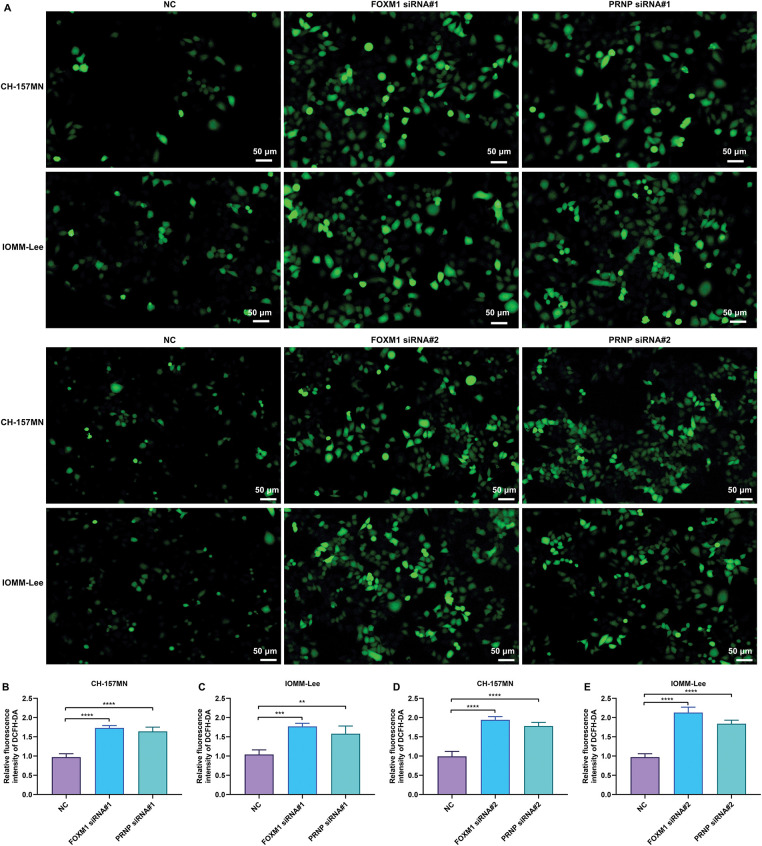
Knockdown of FOXM1 or PRNP induces the excessive accumulation of ROS in meningioma cells. (**A**–**E**) DCFH-DA-labeled immunofluorescence for measuring the intracellular ROS levels in CH-157MN and IOMM-Lee cells under transfection of FOXM1 siRNA#1/#2 or PRNP siRNA#1/#2. Scale bar, 50 μm. **p-value < 0.01; ***p-value < 0.001; ****p-value < 0.0001

The activation of ROS signaling is a critical event of oxidative stress. In FOXM1-, or PRNP-silenced CH-157MN and IOMM-Lee cells, intracellular ROS levels were prominently increased according to the results of DCFH-DA-labeled immunofluorescence (Fig. 6A–E). Silence of FOXM1 and PRNP was thus evidenced to induce the excessive accumulation of ROS in meningioma cells.

## 4 Discussion

To solve the limitations that the WHO grading based upon histopathology usually cannot accurately predict tumor behaviors, several studies have been performed based on gene expression profiling. For example, Thirimanne et al. established the meningioma transcriptomic landscape and demonstrated new subtypes with regionally associated biology and patient outcomes [35]. Through applying systems-biology approaches to transcriptomic data, Zador et al. proposed meta-gene markers for better predicting meningioma recurrence [36]. This study conducted a molecular characterization of oxidative stress-related genes and proposed a novel oxidative stress-based molecular classification in high-grade meningiomas, named cluster 1 and cluster 2. In addition, possible therapeutic vulnerabilities: FOXM1 and PRNP were also discovered and verified. Effective medical treatments are limited for high-grade meningiomas, and novel therapeutic regimens have been hampered by our poor understanding of the biology of high-grade meningiomas [37–39]. Our findings might facilitate the development of novel treatment regimens against this disease.

This study identified 189 up-regulated genes and 737 down-regulated genes in high-grade meningiomas vs. low-grade meningiomas. The differentially expressed genes were notably linked with oncogenic pathways (e.g., cell cycle, cellular senescence, p53, MAPK, PI3K-Akt, Ras, Rap1, cAMP, and FoxO signaling pathways, and pathways in cancers), reflecting their involvement in meningiomas. Oxidative stress-related genes such as AOX1, FOXM1, GPX3, PRNP, and SEPP1 were differentially expressed between high- and low-grade meningiomas. Oxidative stress may contribute to the occurrence and development of many cancer types [40–42], including meningiomas [43,44]. In comparison to cluster 2, adipogenesis, xenobiotic metabolism, bile acid metabolism, ROS pathway, Wnt/beta-catenin signaling, and Notch signaling were remarkably activated in cluster 1, reflecting that meningiomas in cluster 1 might be more aggressive.

Although most meningiomas can be cured by resection, further treatment by radiotherapy may be required, especially for WHO grade 2/3 tumors with an increased risk of recurrence, even after conventional therapies [45]. However, rates of tumor control following adjuvant radiotherapy are highly variable, and poor outcomes are unavoidable. Therefore, novel treatment strategies are urgently needed. Immunotherapy has considerably improved prognostic outcomes for patients with cancer [46,47], including meningiomas [48,49]. Because meningiomas are usually perfusion by the dural branch of the external carotid artery outside the blood-brain barrier, they are possible accessible targets for immunotherapy [50]. Most of the ongoing immunotherapy clinical trials in meningiomas are focused on targeting the programmed cell death protein 1/programmed death ligand 1 (PD-1/PD-L1) axis, such as NCT03173950, NCT03016091, and NCT03279692. In accordance with higher infiltrations of immune and stromal cells and higher expression of classic immune checkpoints: CD86, PDCD1, and LAIR1, cluster 1 meningiomas might be more sensitive to immunotherapy. Therefore, the oxidative stress-based molecular classification might potentially predict the response of patients with meningiomas to immunotherapy.

The large-scale drug screening provided comprehensive insights into the anti-meningioma activities of the US Food and Drug Administration (FDA)-approved drugs, and discovered bortezomib, cytarabine, and talazoparib as novel and effective drugs for the treatment of cluster 1 meningiomas, as well as crizotinib, dabrafenib, docetaxel, foretinib, GSK1904529A, sorafenib, temozolomide, and 5-fluorouracil for cluster 2. A prior pharmacological landscape has identified carfilzomib, omacetaxine, ixabepilone, and romidepsin as possible antineoplastic agents against aggressive meningiomas [51]. Nevertheless, further clinical investigations are required to validate the therapeutic effects of these drugs.

This study established the LogitBoost, randomforest, and SVM models that could differentiate the two oxidative stress-based clusters. The model potentially facilitated the clinical application of the oxidative stress-based molecular classification. In addition, we discovered the three feature genes (APOE, GNPTG, and TSPAN4) identified by LogitBoost, random forest, and SVM machine learning approaches, all of which enabled us to accurately predict the two oxidative stress-based clusters.

Although a few molecular alterations have been discovered in meningiomas, potent therapeutic targets remain limited. Among the key oxidative stress-related genes, FOXM1 and PRNP were experimentally proven to be up-regulated in two human meningioma cells (CH-157MN and IOMM-Lee) vs. one human meningeal cell (HMC). Silence of FOXM1 or PRNP effectively attenuated the growth and migration of meningioma cells as well as accelerated intracellular ROS accumulation. These findings uncovered the potential of FOXM1 and PRNP as therapeutic vulnerabilities of meningiomas. FOXM1 has been evidenced as a critical transcription factor of benign and malignant meningiomas [52,53]. H3K27ac has a specific activation of genes regulated by FOXM1 in malignant meningiomas [54]. In addition, thiostrepton (a FOXM1 inhibitor) in combination with radiotherapy can potently impair the proliferation of malignant meningiomas [55]. This study, for the first time, reported the biological implication of PRNP in meningiomas.

The oxidative stress-based molecular classification might be a promising approach to overcome the limitations of the WHO grading. Several molecular classifications have been proposed. For example, it has been demonstrated that copy number variation (CNV)- and methylation family-based classifications capture clinically more homogenous groups and show a superior accuracy in predicting recurrence risk and prognostic outcomes of meningiomas, outperforming the WHO classification [56,57]. Through integrating somatic CNVs, somatic point mutations, methylation, and mRNA abundance, a consensus molecular classification of meningiomas has been introduced, which has been demonstrated to more accurately predict clinical outcomes than the WHO classification [58]. Another molecular classification has been proposed that can predict response to radiotherapy, discriminating outcomes better than the WHO classification [59]. Different from the existing molecular classifications, the oxidative stress-based molecular classification can predict the response of patients with high-grade meningiomas to immunotherapy. Furthermore, we identified the feature genes to predict the two oxidative stress-based clusters. Therefore, the oxidative stress-based classification we proposed might complement the prior systems. In our future studies, we will further validate the clinical value of the novel oxidative stress-based classification and the two oxidative stress-related genes (FOXM1 and PRNP) in meningiomas.

However, the limitations of this study should be pointed out. Firstly, the clinical value of the oxidative stress-based molecular classification needs to be further validated in prospective multicenter cohorts. Secondly, the feasibility and treatment significance of implementing the oxidative stress-based molecular classification of meningiomas in clinical practice needs to be addressed. Thirdly, the biological roles and specific mechanisms underlying FOXM1 and PRNP in meningiomas warrant in-depth exploration.

## 5 Conclusion

Collectively, the study established a novel oxidative stress-based molecular classification for high-grade meningiomas, which might assist clinical decision-making. Moreover, FOXM1 and PRNP were experimentally evidenced as promising treatment vulnerabilities against meningiomas. The findings may provide a novel insight into previously uncharacterized roles of oxidative stress in meningiomas.

## Supplementary Materials



Figure S2 Identification of differentially expressed genes in high-grade and low-grade meningiomas, and their functional enrichment analysis. (A) Volcano diagram illustrates 189 up-regulated genes, and 737 down-regulated genes in high-grade compared with low-grade meningiomas. Red, up-regulated genes; blue, down-regulated genes; grey, genes without differential expression. (B) Heatmap visualizes the expression of the differentially expressed genes in high-grade and low-grade meningiomas. (C) Heatmap shows the top 20 up- and down-regulated genes in high-grade versus low-grade meningiomas, respectively. (D, E) Upset plots display the main biological processes enriched by up- and down-regulated genes, respectively. (F, G) Upset plots show the main KEGG pathways enriched by up- and down-regulated genes, respectively.

Figure S3 External validation of the oxidative stress-based molecular classification in the GSE74385 dataset. Heatmap shows consensus matrix of meningioma samples at the optimal value (k=2) based on the expression profiles of oxidative stress-related genes.

Figure S4 Full uncropped and unedited western blots.

## Data Availability

All data and materials are published in the manuscript and supplementary materials are available on the journal website.

## Supplementary Materials

The supplementary material is available online at https://www.techscience.com/doi/10.32604/or.2025.066308/s1.

## Abbreviations

WHO: World Health Organization
FOXM1: Forkhead Box M1
PRNP: Prion Protein
ROS: Reactive oxygen species
CD86: Cluster of Differentiation 86
PDCD1: Programmed Cell Death 1
LAIR1: Leukocyte-Associated Immunoglobulin-Like Receptor 1
CNV: Copy number variation
RNA-seq: RNA sequencing
GO: Gene Ontology
KEGG: Kyoto Encyclopedia of Genes and Genomes
PCA: Principal component analysis
LogitBoost: Logistic regression
SVM: Support vector machine
randomforest: Random forest
ROC: Receiver operator characteristic curve
AUC: Area under the curve
RT-qPCR: Reverse transcription quantitative polymerase chain reaction
siRNAs: Small interfering RNAs
NC: Negative control
CCK-8: Cell Counting Kit-8
ROS: Reactive oxygen species
DCFH-DA: 2,7-dichlorofluorescein diacetate
MAPK: Mitogen-activated protein kinases
PI3K: Phosphoinositol-3 kinase
Rap1: Ras-associated protein1
cAMP: Cyclic adenosine monophosphate
FoxO: Forkhead box O
NK: Natural killer
PD-1: Programmed cell death protein 1
PD-L1: Programmed death ligand 1
FDA: Food and Drug Administration
