# Supplementary material for: Identification of a Novel Oxidative Stress-Based Molecular Classification and Treatment Vulnerabilities in WHO Grade 2/3 Meningiomas
Source: Oncol Res. 2025 Sep 26;33(10):2903–21. doi: 10.32604/or.2025.066308 (PMC12494116; doi:10.32604/or.2025.066308)
Supplement: Supplementary file 1 [file OncolRes-33-66308-s001.docx]

**Supplementary files:**

Supplementary figure 1. Identification of differentially expressed genes in high-grade and low-grade meningiomas, and their functional enrichment analysis. (A) Volcano diagram illustrates 189 up-regulated genes, and 737 down-regulated genes in high-grade compared with low-grade meningiomas. Red, up-regulated genes; blue, down-regulated genes; grey, genes without differential expression. (B) Heatmap visualizes the expression of the differentially expressed genes in high-grade and low-grade meningiomas. (C) Heatmap shows the top 20 up- and down-regulated genes in high-grade versus low-grade meningiomas, respectively. (D, E) Upset plots display the main biological processes enriched by up- and down-regulated genes, respectively. (F, G) Upset plots show the main KEGG pathways enriched by up- and down-regulated genes, respectively.

Supplementary figure 2. External validation of the oxidative stress-based molecular classification in the GSE74385 dataset. Heatmap shows consensus matrix of meningioma samples at the optimal value (k=2) based on the expression profiles of oxidative stress-related genes.

Supplementary figure 3. Full uncropped and unedited western blots.

Supplementary table 1. Clinical information of meningiomas in the GSE183653 dataset.

Supplementary table 2. The oxidative stress-related gene set.

Supplementary table 3. The primer sequence of RT-qPCR.

Supplementary table 4. Classification of patients with high grade meningiomas into two oxidative stress-based molecular clusters.
